# Case report: FOXP1 syndrome caused by a *de novo* splicing variant (c.1652+5 G>A) of the *FOXP1* gene

**DOI:** 10.3389/fgene.2022.926070

**Published:** 2022-08-05

**Authors:** Min Chen, Yixi Sun, Yeqing Qian, Na Chen, Hongge Li, Liya Wang, Minyue Dong

**Affiliations:** ^1^ Women’s Hospital, School of Medicine, Zhejiang University, Hangzhou, China; ^2^ Key Laboratory of Reproductive Genetics (Zhejiang University), Ministry of Education, Hangzhou, China; ^3^ Key Laboratory of Women’s Reproductive Health of Zhejiang Province, Women’s Hospital, School of Medicine, Zhejiang University, Hangzhou, China

**Keywords:** FOXP1 syndrome, global developmental delay, intellectual disability, splicing variant, WES, RNA analysis

## Abstract

FOXP1 syndrome is a rare neurodevelopmental disorder characterized by global developmental delay, intellectual disability, and language delay, with or without autistic features. Several splicing variants have been reported for this condition, but most of them lack functional evidence, and the actual effects of the sequence changes are still unknown. In this study, a *de novo* splicing variant (c.1652 + 5 G>A) of the *FOXP1* gene was identified in a patient with global developmental delay, mild intellectual disability, speech delay, and autistic features. Assessed by TA-cloning, the variant promoted the skipping of exon 18 and a premature stop codon (p.Asn511*), resulting in a predicted truncated protein. This variant, that is lacking the forkhead-box DNA-binding domain and nuclear localization signal 2, may disrupt the protein function and thus cause FOXP1 syndrome-related symptoms. Our study extends the phenotypic and allelic spectra of the FOXP1 syndrome.

## Introduction

Foxp1 (forkhead-box protein P1) is a member of the FOX transcription factor family, characterized by the presence of a specific DNA-binding domain known as the forkhead-box (FOX). It contains four main functional domains: an N-terminal glutamine rich region, a zinc finger domain, a leucine zipper domain, and a C-terminal FOX domain (Wang et al., 2003). The Foxp1 protein plays an essential role in neural development (Co et al., 2020), B cell development (Fuxa and Skok, 2007), and monocyte differentiation (Shi et al., 2008). It is encoded by the *FOXP1* gene, which is located on chromosome 3p13 and spans 21 exons. Variants in *FOXP1* are associated with the FOXP1 syndrome (MIM #613670) featured by global developmental delay, intellectual disability, language delay, with or without autistic features (Meerschaut et al., 2017; Siper et al., 2017). Although a dominant negative effect has been reported (Sollis et al., 2016), current evidence holds that haploinsufficiency is the key pathogenic mechanism of this syndrome (Carr et al., 2010; Hamdan et al., 2010; Horn et al., 2010; Le Fevre et al., 2013; Araujo et al., 2015; Myers et al., 2017). Thus far, there are around 141 *FOXP1*-related variants that have been reported in patients based on the HGMD database (Professional release 2022.1), including 49 gross deletions/insertions, 46 missense/nonsense, 31 indels, and 11 splicing variants. Although all of the splicing variants are predicted to affect RNA splicing and produce abnormal transcripts, most predictions have not been confirmed by published transcriptional studies. Therefore, the actual effects of mRNA changes in individuals remain unknown. Hence, caution should be exercised in interpreting these variants, and further functional studies are needed. Herein, one *de novo FOXP1* variant (c.1652 + 5 G>A) was identified in a patient presenting global developmental delay, mild intellectual disability, speech delay, and autistic features. Assessed by an RNA analysis, we demonstrated that this variant promoted the skipping of exon 18 and a premature stop codon (p.Asn511*), and was scored as a pathogenic according to the ACMG guidelines. Our study extends the phenotypic and allelic spectra of the FOXP1 syndrome.

## Materials and methods

### Biological samples

Peripheral blood samples from the patient, his sister, and parents were obtained by venipuncture and collected into a tube containing anethylenediaminetetraacetic acid dipotassium salt (EDTA-K2) (Becton Dickinson and Company, United States). This study was approved by the Ethics Committee of Women’s Hospital, School of Medicine, Zhejiang University, Hangzhou, China (No. IRB-20200216-SC).

### DNA/total RNA extraction, reverse transcription polymerase chain reaction (RT-PCR)

DNA and total RNA were extracted with the QIAamp® DNA Blood Mini Kit (QIAGEN, Germany) and TaKaRaMiniBEST Universal RNA Extraction Kit (TaKaRa, Japan), respectively, according to the manufacturer’s instructions. Complementary DNA (cDNA) was synthesized using the PrimeScript 1st strand cDNA Synthesis Kit (TaKaRa, Japan).

### Chromosome microarray

Genotypes and CNVs were detected by the Affymetrix CytoScan™ HD array (Affymetrix, United States) in accordance with the manufacturer’s instructions. Data were visualized and analyzed with the Chromosome Analysis Suite software (Affymetrix, United States) based on hg19 assembly. Gains ≥500 kb and losses ≥200 kb, containing a minimum of 50 markers in the region were considered as significant.

### Whole exome sequencing

The genomic DNA samples were captured by Agilent SureSelect Human All Exon V6 (Agilent, United States) and sequenced on an Illumina NovaSeq platform(Illumina, United States). BAM alignment file and variant calling were performed using bwa-mem and GATK Haplotype Caller (3.X) from the Sentieon implementation (version sentieon-genomics-201808.03) with the GRCh37/hg19 human genome as a reference. The average sequencing coverage was 120-fold with >92% of the region-of-interest covered at least 20-fold. Variants were filtered through 1000 Genomes, ESP6500, gnomAD databases, and further annotated by AilisNGS® internal pipeline using databases such as ClinVar, the Online Mendelian Inheritance in Man database (OMIM), HGMD, and Ailife internal variant/gene/disease databases.

### TA cloning and sequencing analysis

The fragments containing the variant were amplified using primers IN18-F (5′-GTG​CGT​CAT​AAT​CTT​AGT​CTT​CAC​A-3′) and IN18-R (5′-AAA​ACA​GGA​GGG​ATG​AAA​TGC-3′) for gDNA, and F (5′-CCC​ATT​TCG​TCA​GCA​GAT​ATT​GC-3′) and R (5′-GTA​CAG​GAT​GCA​CGG​CTT​G-3′) for cDNA. The PCR reactions were performed in 50 μl of the total reaction volume containing 50 ng of gDNA or cDNA using 2×GoldStar Best MasterMix (CWBIO, China) at an annealing temperature of 60°C. TA cloning was performed on purified cDNA PCR products using the HieffCloneTM Zero TOPO-TA Cloning Kit (Yeasen, China). Sanger sequencing was conducted on an ABI 3500xL Dx Genetic Analyzer (Applied Biosystems, United States).

### Bioinformatics

The PubMed, OMIM, HGMD, ClinVar, and gnomAD (v2.1.1) databases and the professional version of the Human Splicing Finder system (HSF_Pro) were used to evaluate the pathogenicity of the variants. The mutated protein was predicted by MutPred2.

## Results

**FIGURE 1:**
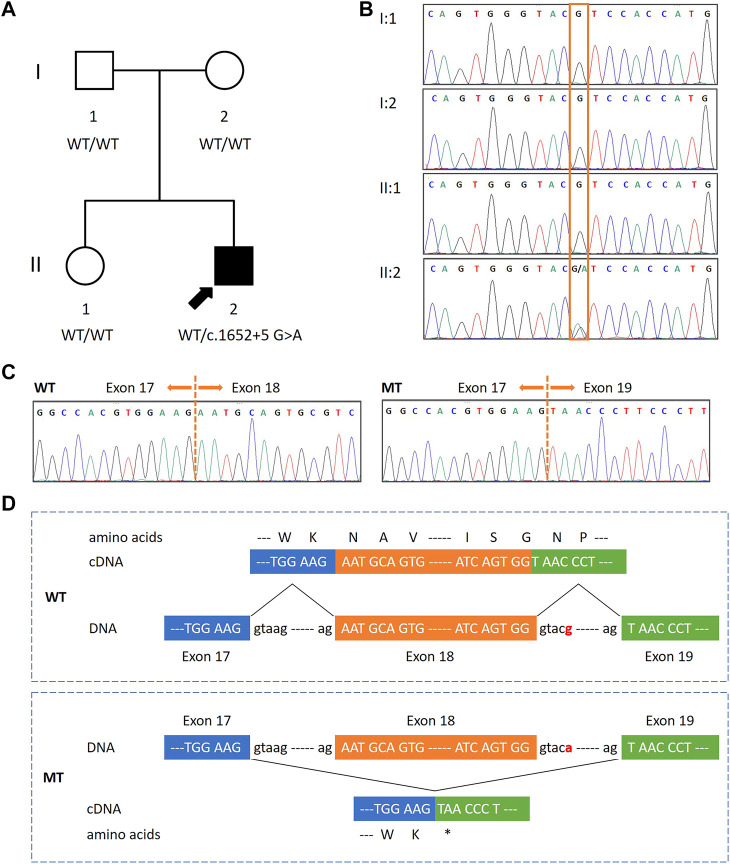
Identification of a *de novo* splicing variant in a patient with global developmental delay, mild intellectual disability and speech delay, and autistic features.**(A,B)** Pedigree diagram and DNA sequencing results of the family. A heterozygous *de novo* splicing variant in *FOXP1* (NM_032682: c.1652 + 5 G>A) was present in the proband (indicated by an arrow). This variant was not found in his parents and sister, who were asymptomatic. The orange box indicates the variant site. **(C)** Sequence electropherograms of *FOXP1* obtained from cDNA from the patient revealed that c.1652 + 5 G>A causes the skipping of exon 18 in mutant (MT) clones as compared to wild-type (WT) ones. **(D)** Schematic diagrams of WT and MT FOXP1. The exon 17, 18, and 19 sequences are represented in upper cases framed by blue, orange, and green boxes, respectively. The splicing variant (designated in red) resulted in the skipping of exon 18 and a new premature stop codon at 511 (p.Asn511*).

The patient was a 29 year old male, the second-born child of non-consanguineous Chinese parents. He had one older unaffected sister and there was no family history of the disease (Figure 1A). He presented global developmental delay, mild intellectual disability, and delayed speech and language development. At the age of 8 months, he was diagnosed with hemolytic anemia and was cured at the age of 5 years. He started to walk at the age of around 3. His hearing and vision were normal. He had attended a specialized educational school and was doing some simple work in a factory. His reading and writing skills were limited. He presented some behavioral problems including autistic features, anxiety, irritability, communication impairments, decreased sociability, and obsessive–compulsive behavior. Recurrent infections of the skin and ears were noted. He also exhibited mild facial dysmorphic features such as blepharophimosis, telecanthus, wide nasal bridge, and irregular dentition. Other findings included sleep disturbance and hyperhidrosis. He did not present seizures or asthma.

His karyotype was normal, and so were the results of the SNP array and the *FMR1* screening (data not shown). Whole exome sequencing (WES) was performed on the patient, which revealed a splice-site variant in intron 18 of the *FOXP1* gene (NM_032682: c.1652 + 5 G>A) in heterozygosis. Sanger sequencing confirmed the presence of the *FOXP1* variant in the patient. It was neither detected in the samples of his parents nor his sister, indicating that the inheritance status was *de novo* (Figure 1B). This variant had neither been reported in the HGMD database nor in the literature. It was annotated as conflicting interpretations of the pathogenicity in ClinVar. Moreover, it was absent from the large population (gnomAD). Located in the fifth nucleotide of intron 18, c.1652 + 5 G>A was predicted to break the donor site and affect the RNA splicing by HSF and MaxEnt. However, no related functional studies have been reported. To demonstrate the molecular consequences of this splice-site variant, we investigated RNA processing by assessing its transcription in peripheral blood mononuclear cells obtained from the patient and his sister. RT-PCR was performed using primers to amplify the cDNA between exons 16 and 20. The direct sequencing of the patient’s RT-PCR products revealed two alleles—a longer one corresponding to the wild-type (WT) sequence and a shorter one corresponding to the mutated (MT) sequence, whereas there was only one unique homozygous WT allele in his sister’s (data not shown). These observations indicated that the aberrant transcript was present in the cDNA from the patient, but not in a healthy control individual. TA cloning followed by sequencing further revealed that the splice-site variant caused the skipping of exon 18 (122 bp) and presumably created a premature stop codon at position 511 (p.Asn511*) (Figures 1C,D). These data revealed that c.1652 + 5 G>A inactivated the splice donor site, which might result in a putative truncated protein. The truncated protein was predicted to have 510 amino acids, and thus was only approximately 75% the size of the functional full-length protein (677 amino acids). More importantly, it would lack the last 167 residues at the C-terminus including a part of the FOX DNA-binding domain and the nuclear localization signal 2 (NLS2). A score of 0.54469 was calculated according to MutPred2, an algorithm for estimating the pathogenicity of stop–gain variants, indicating that the truncated protein could be pathogenic. Overall, these findings showed the pathogenicity of the variant, and thereby it was classified as pathogenic in accordance with the ACMG guidelines.

## Discussion

Eleven splicing variants in the FOXP1 syndrome have been described in the literature (Deciphering Developmental Disorders, 2015; Grozeva et al., 2015; Meerschaut et al., 2017; Siper et al., 2017; Gieldon et al., 2018; Urreizti et al., 2018; Ohashi et al., 2021), and only three of them (c.975–2A>C, c.1428 + 1G>A and c.1428 + 5G>A) have been proved to disrupt splicing by RNA studies. Additionally, ClinVar records 99 intronic variants, involving 25 that are categorized as pathogenic/likely pathogenic, 63 as benign/likely benign, and 11 as uncertain significance/conflicting interpretations of pathogenicity. However, no experimental evidence has demonstrated their impacts on the RNA/protein function except for one variant. Thus, caution is needed when interpreting these disease-causing variants. RNA analysis places an important role in uncovering the impact of splice-site variants, which can be critical in the evaluation of the protein function. In the absence of functional studies, the splicing variant c.1652 + 5 G>A is interpreted as conflicting the interpretations of pathogenicity by ClinVar. Functional characterization in our study provided evidence that the *de novo* heterozygous splicing variant c.1652 + 5 G>A disrupted the donor splice-site and produced an abnormal transcript (p.Asn511*). In comparison, another splicing variant c.1653–2A>T (Bekheirnia et al., 2017) affecting the same intron is located in the acceptor splice-site. Thus, it is predicted to lead to the loss of the acceptor site and is also likely to produce a truncated protein.

The variant p.Asn511* was predicted to yield a C-terminally truncated protein that lacked the characteristic FOX domain and NLS2. The FOX domain is evolutionarily conserved and forms five α-helices (H1-H5) and three β strands (β1-β3) (Benayoun et al., 2011; Siper et al., 2017). The presumed truncated protein in this work probably retains the H1, H2, H4, and β1 but not the H3, H5, β2, and β3. Given that H3, β2, and β3 are regions for DNA recognition and binding (Siper et al., 2017), the discovered variant may interfere with the ability of DNA binding and protein–protein interactions. Located within the FOX domain, NLS2 is also highly conserved and responsible for nuclear import. Consequently, p.Asn511* is unlikely to translocate to the nucleus to perform its function. A similar variant p.Arg525* truncated within the FOX domain displays the mislocalization of protein in cells, loss of the transcription repression activity, and interactions with FOXP1 or FOXP2 (Hamdan et al., 2010; Sollis et al., 2016). By analogy, lacking the FOX domain and NLS2, the truncated protein p.Asn511* is unlikely to retain the normal protein function and thus may result in aberrant subcellular localization, abnormal transcription factor activity, and disrupted protein interactions. The extension of functional studies may lead to a better understanding of biological mechanisms.

By comparison, all C-terminally truncating variants [p.(Leu519Glnfs*10), p.Arg525*, p.(Phe541Leufs*5), and p.Arg544*] and splicing variants (c.1653–2A>T, c.1889 + 5G>T and c.1652 + 5 G>A) present mild to severe developmental delay involving intellectual disability, delayed speech and language development, and motor delay (Hamdan et al., 2010; Bekheirnia et al., 2017; Meerschaut et al., 2017; Zombor et al., 2018; Gao et al., 2019; Braden et al., 2021; Trelles et al., 2021). Other clinical features include autism, facial dysmorphism, behavioral difficulties, recurrent infections, hypotonia, strabismus, and so on. However, the phenotypic variability is wide and there is no significant difference in the severity of phenotypes between these C-terminal variants and others, which is consistent with a previous investigation (Meerschaut et al., 2017). Hemolytic anemia has not been reported in association with *FOXP1*. We hypothesized that it was caused by other genes, but no associated pathogenic variations were observed. Moreover, this symptom was verbally described by the patient’s father but he could not provide any test report. Consequently, it is hard for us to determine the cause without detailed clinical manifestations. Whether this variant might contribute to hemolytic anemia remains an open question; further studies are needed to investigate the effect of FOXP1 on the hematologic system.

In summary, a splicing *de novo* variant in the *FOXP1* gene (c.1652 + 5 G>A) was identified in an adult patient with the FOXP1 syndrome. It was proved to alter splicing and produce an aberrant transcript (p.Asn511*), which may disrupt the protein function and thus cause FOXP1 syndrome-related symptoms. Our study extends the phenotypic and allelic spectra of the FOXP1 syndrome.

## Data Availability

The datasets for this article are not publicly available due to concerns regarding participant/patient anonymity. Requests to access the datasets should be directed to the corresponding author.
